# Daily Changes in Temperature, Not the Circadian Clock, Regulate Growth Rate in *Brachypodium distachyon*

**DOI:** 10.1371/journal.pone.0100072

**Published:** 2014-06-13

**Authors:** Dominick A. Matos, Benjamin J. Cole, Ian P. Whitney, Kirk J.-M. MacKinnon, Steve A. Kay, Samuel P. Hazen

**Affiliations:** 1 Biology Department, University of Massachusetts, Amherst, Massachusetts, United States of America; 2 Molecular and Cellular Biology Graduate Program, University of Massachusetts, Amherst, Massachusetts, United States of America; 3 Molecular and Computational Biology Section, University of Southern California, Los Angeles, California, United States of America; University of Nottingham, United Kingdom

## Abstract

Plant growth is commonly regulated by external cues such as light, temperature, water availability, and internal cues generated by the circadian clock. Changes in the rate of growth within the course of a day have been observed in the leaves, stems, and roots of numerous species. However, the relative impact of the circadian clock on the growth of grasses has not been thoroughly characterized. We examined the influence of diurnal temperature and light changes, and that of the circadian clock on leaf length growth patterns in *Brachypodium distachyon* using high-resolution time-lapse imaging. Pronounced changes in growth rate were observed under combined photocyles and thermocycles or with thermocycles alone. A considerably more rapid growth rate was observed at 28°C than 12°C, irrespective of the presence or absence of light. In spite of clear circadian clock regulated gene expression, plants exhibited no change in growth rate under conditions of constant light and temperature, and little or no effect under photocycles alone. Therefore, temperature appears to be the primary cue influencing observed oscillations in growth rate and not the circadian clock or photoreceptor activity. Furthermore, the size of the leaf meristem and final cell length did not change in response to changes in temperature. Therefore, the nearly five-fold difference in growth rate observed across thermocycles can be attributed to proportionate changes in the rate of cell division and expansion. A better understanding of the growth cues in *B. distachyon* will further our ability to model metabolism and biomass accumulation in grasses.

## Introduction

Primary growth in plants is the product of cell division and elongation. Typically, cell division occurs in the meristems where pluripotent undifferentiated cells are located. Subsequent to cell division, some of the daughter cells replenish the meristem pool while others differentiate and elongate. The elongation process is largely caused by changes in turgor pressure via water intake and storage in the central vacuole accompanied by loosening of the cell wall. This process results in increases in leaf, stem, and root length. Interestingly, growth rate can vary throughout the day, manifested as a diurnal growth rhythm. As with myriad behavioral and physiological traits in plants, growth rhythms are initiated by endogenous mechanisms and external cues [1], [2], [3]. Two external cues implicated in driving growth rhythms are photocycles and thermocycles.

In *Arabidopsis thaliana*, light is perceived by the phytochrome and cryptochrome photoreceptor families that directly interact with growth promoting factors as well as entrain the circadian clock [4], [5], [6]. In continuous light and temperature conditions, *A. thaliana* hypocotyl growth exhibits a rhythm with a peak rate at subjective dusk [7]. These rhythms are abolished in several well-characterized clock mutant lines, indicating this behavior to be the result of circadian clock function [5], [7]. By contrast, under photocycles, growth rhythms peak at dawn rather than dusk [5], [8]. The concurrent effects of the circadian clock that are revealed in the absence of external cues and light regulated events dictate the timing of regulated growth processes [5], [6], [9]. The mechanism underlying this behavior is similar to that of the coincidence of external and internal cues that control photoperiodic flowering in *A. thaliana* [10]. Periodic growth is largely controlled by expression of the growth activating basic helix-loop-helix (bHLH) transcription factors *PHYTOCHROME INTERACTING FACTOR 4* (*PIF4*) and *PIF5*, which are repressed in the early evening by the circadian clock. Subsequent expression results in late night growth that is ultimately repressed by light regulated degradation of PIF4 and PIF5 proteins at dawn the following day [5]. This results in a sharp peak of growth-promoting activity during the late evening. The components and behavior of the circadian clock in numerous plant species appears to be relatively conserved and similar to *A. thaliana*. Unsurprisingly, this may not hold true for photoperiodic mechanisms, considering responses to seasonal day length vary greatly across species [11]. Daily growth rhythms are no exception [3], [12].

Light cycling parameters are clearly important external cues that control growth rhythms. In the presence of photo and thermocycles, eudicot growth oscillations can peak at dawn [8], [13], [14], [15], dusk [16], [17], during the day [18], [19], [20], or during the night [21], depending on the species of plant. Timing of peak growth persists under constant temperature in the presence of photocycles [5], [15], [22] or in constant light with thermocycles [22]. When light and temperature are both held constant, *A. thaliana*, *Ricinus communis*, and *Dendranthema grandiflorum* are known to exhibit a circadian clock regulated growth rhythm [5], [15], [22]. While the timing within a day may vary, photo and thermocycles as well as the circadian clock regulate daily growth rhythms in eudicot species.

The mechanisms that regulate rhythmic growth in grasses are likely distinct from eudicots. Rapid growth during the day relative to the night has uniformly been observed in numerous grasses [23], [24], [25], [26], [27], [28]. However, *Z. mays* and *O. sativa* and possibly other grasses exhibit arrythmic growth of above ground tissues in constant conditions [15]. Under constant temperature, growth oscillations still occur under photocycling conditions in *Z. mays* and *Fescue arundinacea*, where growth rate peaks during the night, but these rhythms are much less robust than those observed under photocycles and thermocycles together [15], [29]. Growth in both species under thermocycles and continuous light mimics the presence of both cues, implicating temperature as the major contributor to cyclic growth [15].

Cell division and elongation rates increase with temperature in grass leaves [15], [30], [31], [32], suggesting that temperature dependent enzyme activity coordinates the networks that influence growth. Another possible mechanism of coordination may be through a common regulator that senses temperature change and initiates a signal transduction cascade resulting in diurnally controlled growth. This latter possibility appears to be the more plausible model considering the variable activity of growth associated enzymes and photosynthetic rates measured within *A. thaliana*, *Z. mays*, and *O. sativa* [32]. Regardless, temperature and not light or the circadian clock is the most important cue influencing daily growth rhythms in grasses.

It is essential to translate mechanistic understanding of growth regulation to energy crops where total biomass accumulation is the most vital attribute. Currently, several grasses are in various stages of development as energy crops including *Panicum virgatum*, *Miscanthus spp*., *Z. mays*, *S. bicolor*, and prairie grass mixtures [33], [34], [35], [36], [37]. Several relevant differences between these species and the model system *A. thaliana* dictate that one or more grass species should serve as the focus of in depth study. Energy crops have many features unfavorable to laboratory research, namely large and often polyploid genomes, large stature, long lifecycles, and cumbersome genetic and genomic tools. In contrast, *Brachypodium distachyon* exhibits many model system attributes including a sequenced genome, notable transformation efficiency, small stature, and a rapid life cycle [38], [39]. Additionally, due to its short stature and rapid growth, *B. distachyon* makes an excellent subject for image-based assays of growth. These assays offer numerous benefits over more traditional growth measurement techniques (e.g. direct measurement, or recording via a linear variable displacement transducer [40]), as it is non-invasive, inexpensive, and unbiased. Recently, time-lapse photography of leaf, root, and stem with high-resolution CCD cameras has led to better understanding of factors influencing dynamic changes in morphology [15], [41], [42], [43]. Plants lack the ability to sense wavelengths above far-red (>800 nm); therefore, infrared lighting can be used to illuminate plants in otherwise complete darkness [5], [15], [42], a necessity for measurements in diurnal conditions. Using these methods, total length or area can be calculated for each image within the time-lapse sequence. Indeed, numerous phenomic platforms have both automated image acquisition and analysis [44], [45], [46]. Here we use an image-based assay to investigate the effects of light, temperature, and the circadian clock on daily growth rhythms in *B. distachyon*.

## Materials and Methods

### Plant Material

Dry seed of *B. distachyon* accession Bd21-3 was imbibed and stratified in a wet paper towel at 6°C for sixteen days. Seeds were then sown towards one edge of 10 cm pots containing potting mix (#2; Conrad Fafard Inc., Agawa, MA, USA). Half of the soil was covered with 0.508 mm thick infrared absorbing paper followed by 0.254 mm infrared light absorbing paper (Edmund Optics; Barrington, NJ, USA). Plants were cultivated and imaged in a Percival model CU36L6 Growth Chamber (Percival Scientific, Perry, IA, USA). Light conditions were 62 to 74 µmol of photons⋅m^−2^⋅s^−1^ unless otherwise indicated as bright light, which was 300 to 340 µmol of photons⋅m^−2^⋅s^−1^ in a Conviron model A1000 Growth Chamber (Conviron, Winnipeg, MB, Canada). The hot cold temperature parameters correspond to 28±0.6°C and 12±0.6°C, respectively. Percival conditions were monitored and recorded using a HOBO U12 Data Logger (Onset Computer Corporation, Bourne, MA, USA).

### Imaging System

**Figure 1:**
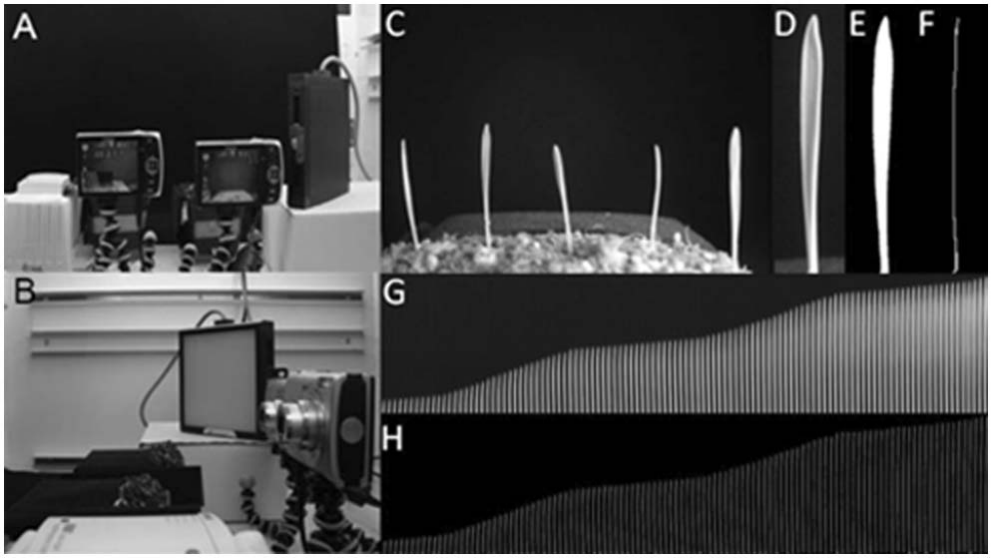
Leaf growth imaging system and leaf length analysis. (**A–B**) Two CCD cameras filtered to exclusively image the infrared spectrum each placed 50 mm from a (**C**) pot illuminated by an 880 nm infrared light. (**D**) Each leaf in each frame was (**E**) converted to a binary image and (**F**) skeletonized for automated length measurement. Visualization of sequentially cropped (**G**) leaf images and (**H**) skeletonization from a time course experiment. The experiment was performed at least twice with similar results.

Time-lapse photography was conducted using modified Canon SD870 IS cameras (Canon, Lake Success, NY, USA). This 8 megapixel digital camera has a 3.8x wide-angle optical image stabilized zoom and a 28 mm lens. The camera was modified with a filter in order to capture only the infrared spectrum ranging from 750 to 1000 nm (Life Pixel; Mukilteo, WA, USA). The Canon Hack Development Kit (CHDK) advanced operating system file on an 8 GB standard SHDC memory card along with a DiskBoot BIN file allowed the Canon SD870 IS camera to boot into the CHDK operating system via the firmware enhancement option found in the camera operating system menu. A script was adapted to provide the camera with time-lapse capability featuring an internal tick clock, fixed focus, camera flash disablement, and infinite capturing ability. The script was used to capture an image every 30 minutes with a −2 exposure with macro and black and white settings enabled. The cameras were placed 50 mm away from the pots (Fig. 1A). A 100×100 mm LED backlight emitting 880 nm infrared light (Edmund Optics, Barrington, NJ, USA) was placed 90 mm away from the pots to the right of the camera to illuminate the growing plants (Fig. 1A–B). The cameras and LED backlight were placed on a horizontal plane as the soil surface (Fig. 1B).

### Image Analysis

The images were compiled into movies using Quicktime Pro (Apple, Cupertino, CA, USA) at a rate of 24 frames per second and captions were added to the movies using Windows Live Moviemaker (Microsoft, Redmond, WA, USA). ImageJ was used to analyze the image sequences [47]. The images were cropped such that only the first or the second true leaf was entirely visible in a given window without any interference of neighboring leaves, during a period of time that the selected leaf was growing, i.e., just after emergence from the sheath, but before bending and leaf maturation occurred. During this period of elongation, leaf growth was predominantly vertical, so 3-D projection artifacts were not expected to confound the data. The cropped leaf image was converted to a binary image (Fig. 1C–E). Any gaps present in the foreground due to binary thresholding were filled, and two to three morphological erosions were subsequently performed (Fig. 1E). Afterwards, the binary image was skeletonized and the area of this skeleton was recorded as an approximation of leaf length (Fig. 1F). This process was done for every frame in which the leaf was visible (Fig. 1G–H). Length was calibrated against an image taken with a ruler after each imaging experiment. Growth rate was calculated as the leaf length measurement minus the leaf length from the previous measurement and plotted as the average of every two hours.

### Gene Expression Analysis

Freshly harvested Bd21 seeds were de-hulled by removing the lemma and then soaked in water for 2 hours. Seeds were sterilized in 10% bleach, 0.1% TritonX-100 solution for 5 minutes and then rinsed 3 times with sterile water. Sterilized seeds were then stratified at 4°C in the dark for 2 days, before being sown onto sterile 1X strength MS substrate (with 0.6% Agar) in a Magenta GA-7 vessel (Sigma Aldrich, St. Louis, MO, USA), and kept in the dark for 3 days at room temperature. Germinated epicotyls were then transferred to incubators (Percival Scientific, Perry, IA, USA) under diurnal temperature and light cycles (LDHC; 28°C 12 hr days with 50 µmol·m^−2^·s^−1^ fluorescent white light, 12°C 12 hr nights). After 10 days of diurnal entrainment, half of the seedlings were transferred to continuous light conditions (LLHH; 28°C 24 hour days with 50 µmol·m-2·s^–1^ fluorescent white light) for 12 hours beginning at CT12, just before dusk. Leaf samples were taken beginning at ZT24 for the LLHH samples (CT0 for the LDHC samples) at 3.5 hour intervals for 2 days for a total of 14 samples per time course.

For sampling, a single leaf was carefully harvested from each plant such that the sheath area was kept intact, immediately freezing 3–4 leaves from separate plants in liquid nitrogen. This tissue was then ground to a fine powder using metal ball bearings and a grinder (Retsch, Haan, Germany). Total RNA was then extracted from the frozen samples using the Qiagen RNeasy Plant Mini Kit (Qiagen, Dusseldorf, Germany) according to the manufacturer instructions. mRNA was then isolated with 2 rounds of polyA selection using a Dynabeads mRNA purification Kit (Life Technologies, Carlsbad CA) according to the manufacturer’s instructions. The cDNA was synthesized and quantified as previously described [48] with the following primers: *BdCCA1* (Bradi3g16515), forward (F), 5′- AGCTTGGCAGCGCATAGAAGAG -3′; reverse (R), 5′- AGCATGGCTTCTGATTTGCACAG -3′; *BdGI* (Bradi2g05226), F, 5′- CCTGTCGAATCTGCTGAAGTGC -3′; R, 5′- ACTGTTCAAGATGTCGCGAAGGAC -3′; *BdLUX* (Bradi2g62067), F, 5′- AGGCAAGAGATGGTGATGCTCTG -3′; R, 5′- AGCAGAGCAAAGACACACACAGG -3′; *BdACT7* (Bradi3g30710), F, 5′- CCTGAAGTCCTTTTCCAGCC -3′; R, 5′- AGGGCAGTGATCTCCTTGCT -3′. The *ACT7* gene was used as a normalization controls [49]. BdGI, BdCCA1, and BdLUX were identified as having the greatest amino acid sequence similarity to *A. thaliana* CCA1 (At2g46830), GI (AT1G22770), and LUX (At3g46640) proteins [50], [51].

### Division Zone Analysis

Plants were entrained in LDHC for 25 days and transferred to LLHC. Whole plants were removed from soil at ZT4 and ZT16 and immediately frozen in liquid N_2_. The emerging leaf on the largest tiller attached to the third node was bleached in boiling methanol for 5 to 10 minutes. Whole leaves were then washed in 1% periodic acid for 40 minutes followed by an overnight treatment of Schiff reagent and propidium iodide (100 mM Na_2_S_2_O_5_ and 0.15 N HCl; propidium iodide to a final concentration of 100 µg/mL). The abaxial surface of the leaf starting at the ligule was visualized using a Leica LSM700 confocal microscope. Files of cells that were located midway between two veins were imaged from the ligule towards the leaf tip. The last newly divided cell determined the distal end of the division zone.

## Results

### Growth Increases Under Warm Light Conditions

**Figure 2:**
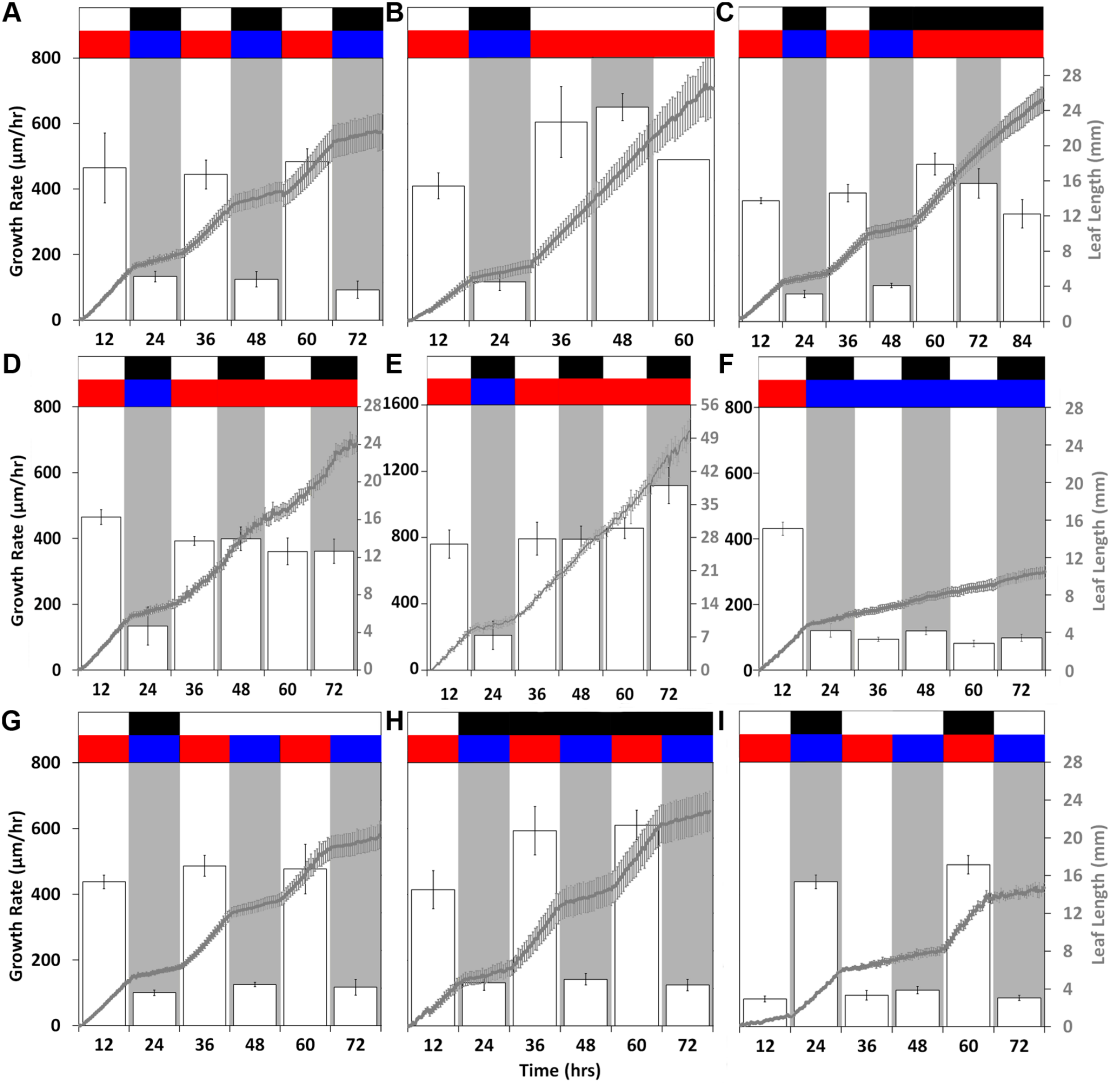
Leaf growth under different regimes of light and temperature. Leaf length (gray) and growth rate (white bars with black lines) of the first leaves. All plants were entrained and imaged under (**A**) photo and thermocycles and then transferred to (**B**) constant 28°C light or (**C**) dark conditions; (**D, E**) 28°C or (**F**) 12°C conditions with continued photocycles; (**G**) constant light or (**H**) dark conditions with continued thermocycles; or (**I**) 12°C days and 28°C nights. Leaf length data are means ± SEM, n = 6–7. The experiments were performed at least twice with similar results.

We first characterized growth under diurnal conditions of 12 hr light, 28°C and 12 hr dark, 12°C (LDHC) by measuring leaf length (Fig. S1A, 2A). Growth rhythms were clearly observed, oscillating with a 24 hr period. Growth rate dramatically increased within the first hour of the day and the growth rate decreased rapidly following the transition from light to dark (Fig. 2A, Movie S1–2). The length of the first leaf increased by an average of 5.3 mm during 28°C light conditions and 1.5 mm during 12°C dark conditions (Table 1). Accordingly, the average growth rate was considerably greater during simulated day conditions (432 µm/hr) than nighttime conditions (120 µm/hr).

**Table 1:** Summary of total leaf growth ± s.e.m of each twelve-hour period of nine different conditions.

Condition†	Day	Night	Fold change
	^_______________________________________________________________________^mm^______________________________________________________________________^		
LDHC	5.29±.52	1.54±.18	3.4
LLHH	6.61±.46	6.73±.42	1.0
DDHH	4.37±.46	4.67±.51	0.9
LDHH	4.52±.17	4.29±.16	1.1
LDHH‡	9.07±.51	10.47±.91	0.9
LDCC	0.78±.09	1.13±.12	0.7
LLHC	5.26±.71	1.44±.20	3.7
DDHC	6.55±.65	1.61±.19	4.1
LDCH	1.02±.15	5.27±.19	0.2

† L, light; D, dark; H, 28°C; C, 12°C.

‡ Bright light as described in the methods.

### Growth is Arrhythmic under Constant Light or Constant Dark Conditions

To elucidate the effect of the circadian clock on growth rhythms, we measured leaf length under constant light and temperature conditions. After entrainment in 4 days of LDHC, growth conditions were changed to constant light and 28°C, LLHH (Fig. S1B). Growth remained constant, similar to what was observed during daytime conditions in the diurnal time course (Fig. 2B, Movie S3–4). Leaf length increased by an average of 6.6 mm during subjective day and 6.7 mm during subjective night (Table 1). Likewise, growth rate was continuous with a subjective day average of 589 µm/hr and subjective night average of 603 µm/hr (Fig. 2B). To test if constant light drives growth and masks a circadian clock regulated growth oscillation, we measured leaf length under constant dark at 28°C (DDHH), following LDHC entrainment (Fig. S1C). Initially, growth persisted similar to daytime conditions in LDHC (Fig. 2C, Movie S5–6). Leaf length increased by an average of 4.4 mm during subjective day and 4.7 mm during subjective night (Table 1). Within the course of the first day of constant darkness, growth rate began to diminish to a rate of 250 µm/hr, likely due to reduced photosynthate availability. The average growth rate in DDHH was 364 µm/hr during the subjective day and 389 µm/hr during the subjective night. As might be expected, growth was slower in DDHH than in LLHH. Nonetheless, the similar and constant growth rate observed under 28°C regardless of light conditions suggests that the circadian clock does not influence growth rate in *B. distachyon*.

### Growth is Marginally Affected or Unaltered by Photocycles and Constant Temperature

We next investigated the effects of photocycles on growth rhythms. Following LDHC entrainment, growth conditions were changed to a constant temperature of 28°C with 12 hr light and 12 hr dark, LDHH (Fig. S1D). Growth increased in the light by an average of 4.5 mm and 4.3 mm in the dark, similar to what was observed during daytime condition in the diurnal time course (Table 1, Fig. 2D, Movie 7–8). Accordingly, the average growth rate was slightly greater during subjective day (389 µm/hr) than in subjective night (372 µm/hr) (Fig. 2D). Even though growth in the dark and light periods in LDHH were similar, the growth rate diminished gradually in the dark, albeit considerably less than the decrease observed in 12°C dark conditions. While plants grown under similar conditions, but brighter light did not exhibit a growth rhythm, the growth rate was 2-fold greater (Fig. S1E, 2E, Movie 9–10, Table 1).

To further elucidate the effects of light on growth, we measured leaf length under a constant temperature of 12°C with 12 hr light and 12 hr dark, LDCC (Fig. S1F). Under these conditions, growth was maintained at a rate similar to that observed in the nighttime conditions of LDHC conditions (Fig. 2F, Movies S11–S12). Leaf length increased by 0.8 mm with an average rate of 65 µm/hr at subjective day by 1.1 mm with an average of 95 µm/hr during subjective night (Table 1, Fig. 2F). No oscillations were observed and total growth was much slower during LDCC overall (80 µm/hr) than LDHH (371 µm/hr) (Fig. 2E, F). This slight or apparent lack of effect by photocylces on diurnal growth rhythms of *B. distachyon* seedlings strongly suggests little or no impact of light or photoreceptor-related signaling on leaf growth rhythms.

### Thermocycles Alone are Sufficient to Induce Rhythmic Growth

Considering the waveform properties observed in LDHC were not emulated in the four constant temperature conditions, LLHH, DDHH, LDHH, or LDCC, we measured leaf growth in constant light with a 12 hr 28°C and 12 hr 12°C thermocycle, LLHC (Fig. S1G). Growth was similar to that observed in LDHC (Fig. 2G, Movie S13–14). Leaf length increased by an average of 5.4 mm during the day and 1.5 mm during the subjective night (Table 1). Accordingly, the growth rate was considerably greater during the day (453 µm/hr) than during nighttime condition (124 µm/hr). Similar to LDHC, growth rate increased gradually through the course of the day, peaking 3 hours prior to subjective night (Fig. 2G). To test if temperature could drive growth rhythms in the absence of light, we measured leaf length under constant darkness with a 12 hr 28°C and 12 hr 12°C thermocycle, DDHC (Fig. S1H). As in LLHC, growth patterns were similar to LDHC (Fig. 2H, Movie S15–16). The length of the first leaf increased by an average of 6.5 mm during the subjective day and 1.8 mm during subjective night. The average subjective day and night growth rate was 542 and 152 µm/hr, respectively. While growth rate was faster during DDHC than LLHC, the peak rate occurred at three hours before subjective dusk in both conditions (Fig. 2G, H). Taken together, these results strongly suggest that thermocycles can drive rhythmic growth.

We next tested the effect of inverting photo and thermocycle treatments. Following growth in LDHC for three days, thermocycles were inverted to 12°C during the light period and 28°C during the dark period, thus LDCH (Fig. S1I). Under these conditions, leaf length increased by 5.3 mm in response to 28°C dark conditions and only by 1.1 mm in 12°C light conditions (Table 1, Fig. 2I, Movie S17–18). Appropriately, growth rate was greater at the daytime temperature in the dark (431 µm/hr) than the nighttime temperature in the light (90 µm/hr). Interestingly, peak growth rate occurred 3 hr into the 28°C dark period similarly to what was seen in LDHH and LDCC (Fig. 2D, 2E, 2I). Also, amplitude changes during day night transitions were sharp and occurred within 5 hr evoking what is typically seen when conditions are changed from 28°C to 12°C in LDHC, LLHC, and DDHC (Fig. 2F, 2G, 2H).

### In Constant Conditions, Putative Clock Gene Expression Exhibits Circadian Behavior

**Figure 3:**
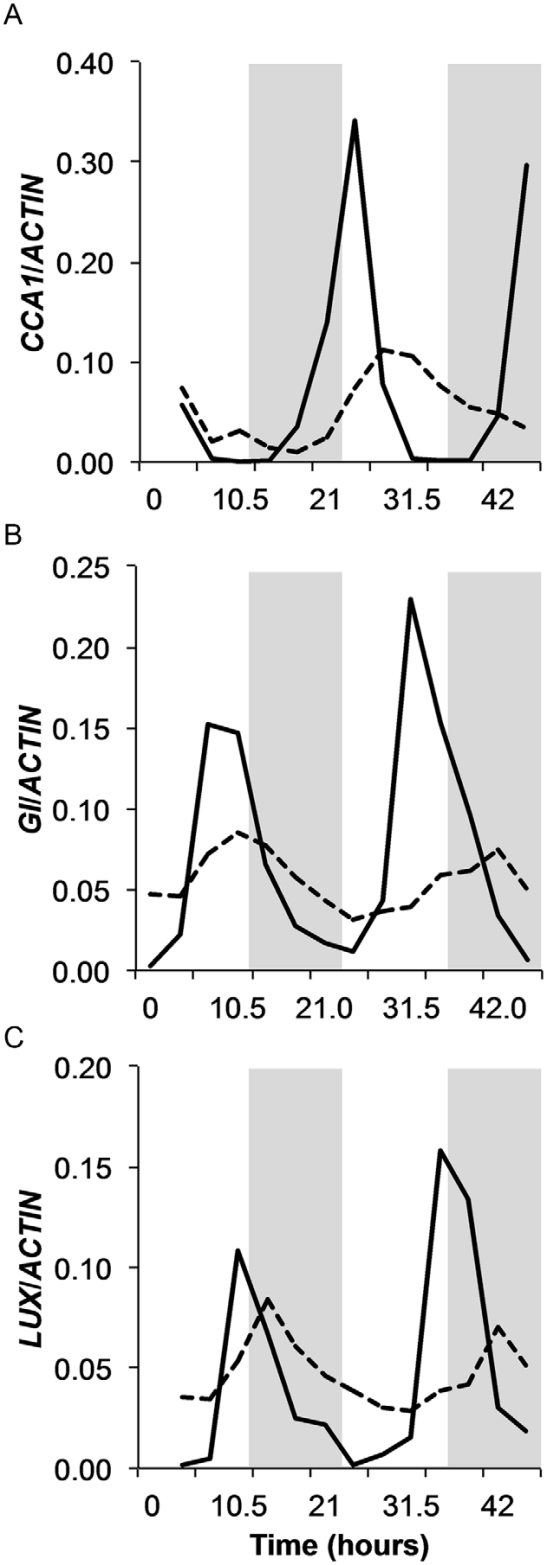
*Brachypodium distachyon* exhibits both diurnal and circadian clock regulated gene expression. Relative transcript abundance was quantified for (A) *BdCCA1*, (B) *BdGI*, and (C) *BdLUX* in leaves sampled every 3.5 hours for two days from plants grown under thermo and photocycles (solid line) or constant conditions (dashed lines).

Following the observation of the absence of growth rhythms in constant conditions, we assayed for circadian clock regulated gene expression. Epicotyls of seedlings grown under combined photo and thermocyles followed by constant light and 28°C conditions were collected every 3.5 h over two days. The relative abundance of putative clock genes, *BdCCA1*, *BdGI*, and *BdLUX*, was assayed by qRT-PCR. The levels of all three transcripts cycled under both regimes with a phase similar to their *A. thaliana* orthologs (Fig. 3A–C). Under LDHC, *BdCCA1* transcript level was greatest at dawn and slightly later in LLHH (Fig. 3A). Peak expression of *BdGI* occurred later in the day at ZT7 in LDHC with a slight phase delay in LLHH (Fig. 3B). *BdLUX* peaked at around ZT11 in LDHC with an average phase of ZT16 in LLHH (Fig. 3C). These results are a clear indication of circadian clock function in *B. distachyon*.

### Leaf Meristem Size is not Affected by Thermocycles

**Figure 4:**
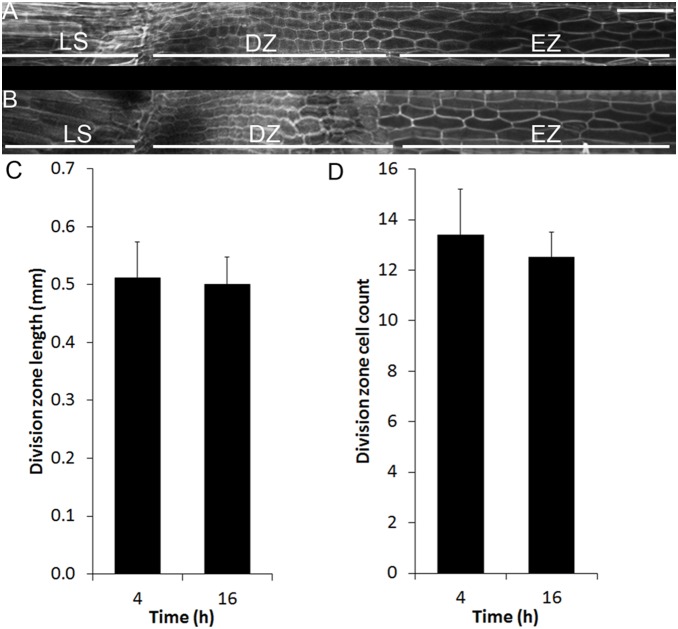
Changes in temperature-regulated growth were not associated with changes in *Brachypodium distachyon* leaf division zone size. The base of elongating leaves grown in constant light with thermocycles sampled during the day (A) and night (B) stained with propidium iodide. The length (C) and number (D) of cells in the leaf division zone. Images were taken using confocal microscopy. LS, leaf sheath; DZ, division zone; EZ, elongation zone. Bars = 50 µm.

Leaf expansion in grasses is the cumulative effect of individually expanding cells that define the region distal of the division zone. The number of cells and rate of cell elongation determines leaf expansion rate. To test if the number of dividing cells varied with rate, we measured the size of the division zone across thermocycles by microscopic analysis (Fig. 4A–B). Seedlings were grown in constant light and thermocycles and the leaf attached to the third node was sampled at ZT4 and ZT16. This developmental stage occurred 23 to 28 days after germination at a growth stage approximate to 33 on the BBCH-scale for cereals [52]. The division zone length at 28°C, 0.51±0.06 mm, was very similar to leaves sampled at 12°C, 0.50±0.05 mm (Fig. 4C). The number of cells in a linear file in the division zone was also not significantly different (Fig. 4D). Therefore, the five-fold differential growth rate observed between 12 and 28°C was not caused by a greater number of expanding cells.

## Discussion

Growth rhythms of *B. distachyon* under diurnal conditions were typical of other grass species grown both in the field and controlled environments with a peak growth rate observed during the day [15], [25], [28]. In the absence of external cues, oscillations were lost, demonstrating that the circadian clock had no influence on daily growth rhythms. This observation has also been noted in both *Z. mays* and *O. sativa* [15]. Interestingly, the lack of growth rhythms in constant conditions is not due to an absence of a circadian clock in grasses. Gene expression and mutant analysis have implicated several orthologs to known *A. thaliana* clock genes to have a similar function is grasses including *ELF3* in barley and rice [53], [54], [55], [56], the *PRR* gene family in rice, barley and sorghum [57], [58], [59], *GI* in rice and wheat [60], [61], and *LUX* in barley [62]. Indeed, the flowering time phenotype of the *A. thaliana gi* mutant can be rescued by *BdGI* [50]. While many of the constituents and component behaviors are conserved among angiosperms, a means of clock regulated leaf expansion is not present in *B. distachyon*.

Our observations showed weak rhythms in low light photocycles and constant temperature conditions with a peak growth rate at dusk, an opposite phase than what was observed in *Z. mays* and *F. arundinacea* [15], [29]. It has been suggested that water availability may have played a role as evaporative demand increases over the course of a day and decreases at night; growth responds inversely in *Z. mays* [15], [31]. In our study, growth was fastest during the subjective day, ruling out this potential explanation for *B. distachyon*. Even though growth was arrhythmic in higher light photocycles and constant temperature conditions, the absolute growth rate was twice that observed under lower light condition. Temperature is clearly a major driver of growth rate in *B. distachyon*, a result also observed in other grasses such as *Z. mays*, *O. sativa,* and *F. arundinacea* [15], [29], [30], [32]. Growth oscillations during LLHC and DDHC were very similar to the ones observed during the diurnal time course suggesting that temperature is sufficient in driving growth oscillations. Furthermore, growth rates under LDHH, LLHH, and DDHH conditions were very similar to those observed during the day in LDHC conditions; while rates observed in LLCC more closely resemble growth at night in LDHC. Taken together, growth rate appears to be influenced primarily by thermocycles alone. The importance of the effect of temperature was more evident when photo and thermocycles were inverted and growth was always greatest at 28°C regardless of light conditions. The stems of two eudicot species, *D. grandiflorum* and *Solanum lycopersicum*, have been shown to exhibit similar behavior in inverted photo and thermocycles [22], [63]. To our knowledge, this observation has not been made previously in grasses.

Organ and organism size varies due to differences in cell number, size, or both. The same holds true for growth rate, which is a function of cell elongation rate and the number of growing cells. Perturbation of the gibberellin (GA) phytohormone pathway in maize results in altered leaf length in maize [64]. Overexpression of AtGA20-oxidase1 in maize results in an enlarged division zone and increased leaf growth. Conversely, the maize *dwarf3* mutant that almost completely lacks bioactive GA1 has a significantly shorter leaf growth rate, which was a product of a smaller division zone with fewer cells than wild type. The faster growing leaves had a larger number of dividing cells that fully explained the increased growth rate. Here, wild-type *B. distachyon* exhibited consistent division zone size in leaves expanding at warm temperatures at a rate of ∼500 µm/hr or colder temperatures at a rate of ∼100 µm/hr. Leaf epidermal cell length remains fairly constant in maize grown at constant temperatures ranging from 13 and 34°C or thermocycles of 25°C/4°C or 25°C/18°C [65], [66]. Considering the likelihood of static final cell lengths, we expect that a proportional increase in both elongation and cell division rate account for the rapid rate changes observed in *B. distachyon* leaf expansion. Increased cell displacement from the division zone can account for the constant meristem size.

The effects of light, temperature, and the circadian clock on the daily growth rhythms of *B. distachyon* and other grasses are distinct from eudicots. This may result from differences in the exposure of the apical meristem to temperature [2]. The eudicot growth zone is typically located apically and relatively exposed to the environment. In grasses, the growth zone is often surround by leaf sheathes. As it is not exposed to light, the grass growth zone is not photosynthetically active and thus growth control by the circadian clock may not be necessary [1]. An intriguing question is whether growth rate in grasses is a passive reaction sensitive to temperature either through changes in enzyme activity or biomechanics of stress physiology or a process actively regulated by a signal transduction cascade downstream of a temperature sensor. There is likely a more complicated mechanism at work here involving multiple growth regulatory mechanisms, which remain to be characterized.

## Supporting Information

Figure S1 **Growth chamber conditions.** All plants were grown under **(A)** photo and thermocycles and then transferred to **(B)** constant 28°C light or **(C)** dark conditions; **(D)** 28°C or **(F)** 12°C conditions with continued photocycles; **(E)** 28°C conditions with continued bright light photocycles; **(G)** constant light or **(H)** dark conditions with continued thermocycles; or **(I)** 12°C days and 28°C nights.
(TIF)

Movie S1 **Infrared time-lapse photography of Brachypodium distachyon seedlings growing in 12 hr light, 28°C and 12 hr dark, 12°C.** Conditions are noted in each frame.
(WMV)

Movie S2 **Infrared time-lapse photography of** ***Brachypodium distachyon*** **seedlings growing in 12 hr light, 28°C and 12 hr dark, 12°C.** Conditions are noted in each frame.
(WMV)

Movie S3 **Infrared time-lapse photography of** ***Brachypodium distachyon*** **seedlings growing in 12 hr light, 28°C and 12 hr dark, 12°C followed by constant light and 28°C.** Conditions are noted in each frame.
(WMV)

Movie S4 **Infrared time-lapse photography of** ***Brachypodium distachyon*** **seedlings growing in 12 hr light, 28°C and 12 hr dark, 12°C followed by constant light and 28°C.** Conditions are noted in each frame.
(WMV)

Movie S5 **Infrared time-lapse photography of** ***Brachypodium distachyon*** **seedlings growing in 12 hr light, 28°C and 12 hr dark, 12°C followed by constant dark and 28°C.** Conditions are noted in each frame.
(WMV)

Movie S6 **Infrared time-lapse photography of** ***Brachypodium distachyon*** **seedlings growing in 12 hr light, 28°C and 12 hr dark, 12°C followed by constant dark and 28°C.** Conditions are noted in each frame.
(WMV)

Movie S7 **Infrared time-lapse photography of** ***Brachypodium distachyon*** **seedlings growing in 12 hr light, 28°C and 12 hr dark, 12°C followed by photocyles and constant 28°C.** Conditions are noted in each frame.
(WMV)

Movie S8 **Infrared time-lapse photography of** ***Brachypodium distachyon*** **seedlings growing in 12 hr light, 28°C and 12 hr dark, 12°C followed by photocyles and constant 28°C.** Conditions are noted in each frame.
(WMV)

Movie S9 **Infrared time-lapse photography of** ***Brachypodium distachyon*** **seedlings growing in 12 hr bright light, 28°C and 12 hr dark, 12°C followed by photocycles and constant 28°C.** Conditions are noted in each frame.
(WMV)

Movie S10 **Infrared time-lapse photography of** ***Brachypodium distachyon*** **seedlings growing in 12 hr bright light, 28°C and 12 hr dark, 12°C followed by photocycles and constant 28°C.** Conditions are noted in each frame.
(WMV)

Movie S11 **Infrared time-lapse photography of** ***Brachypodium distachyon*** **seedlings growing in 12 hr light, 28°C and 12 hr dark, 12°C followed by photocycles and constant 12°C.** Conditions are noted in each frame.
(WMV)

Movie S12 **Infrared time-lapse photography of** ***Brachypodium distachyon*** **seedlings growing in 12 hr light, 28°C and 12 hr dark, 12°C followed by photocycles and constant 12°C.** Conditions are noted in each frame.
(WMV)

Movie S13 **Infrared time-lapse photography of** ***Brachypodium distachyon*** **seedlings growing in 12 hr light, 28°C and 12 hr dark, 12°C followed by constant light and thermocycles.** Conditions are noted in each frame.
(WMV)

Movie S14 **Infrared time-lapse photography of** ***Brachypodium distachyon*** **seedlings growing in 12 hr light, 28°C and 12 hr dark, 12°C followed by constant light and thermocycles.** Conditions are noted in each frame.
(WMV)

Movie S15 **Infrared time-lapse photography of** ***Brachypodium distachyon*** **seedlings growing in 12 hr light, 28°C and 12 hr dark, 12°C followed by constant dark and thermocycles.** Conditions are noted in each frame.
(WMV)

Movie S16 **Infrared time-lapse photography of** ***Brachypodium distachyon*** **seedlings growing in 12 hr light, 28°C and 12 hr dark, 12°C followed by constant dark and thermocycles.** Conditions are noted in each frame.
(WMV)

Movie S17 **Infrared time-lapse photography of** ***Brachypodium distachyon*** **seedlings growing in 12 hr light, 28°C and 12 hr dark, 12°C followed by inverted photo and thermocycles.** Conditions are noted in each frame.
(WMV)

Movie S18 **Infrared time-lapse photography of** ***Brachypodium distachyon*** **seedlings growing in 12 hr light, 28°C and 12 hr dark, 12°C followed by inverted photo and thermocycles.** Conditions are noted in each frame.
(WMV)
