# Supplementary figures and images for: Daily Changes in Temperature, Not the Circadian Clock, Regulate Growth Rate in Brachypodium distachyon
Source: PLoS One. 2014 Jun 13;9(6):e100072. doi: 10.1371/journal.pone.0100072 (PMC4057399; doi:10.1371/journal.pone.0100072)

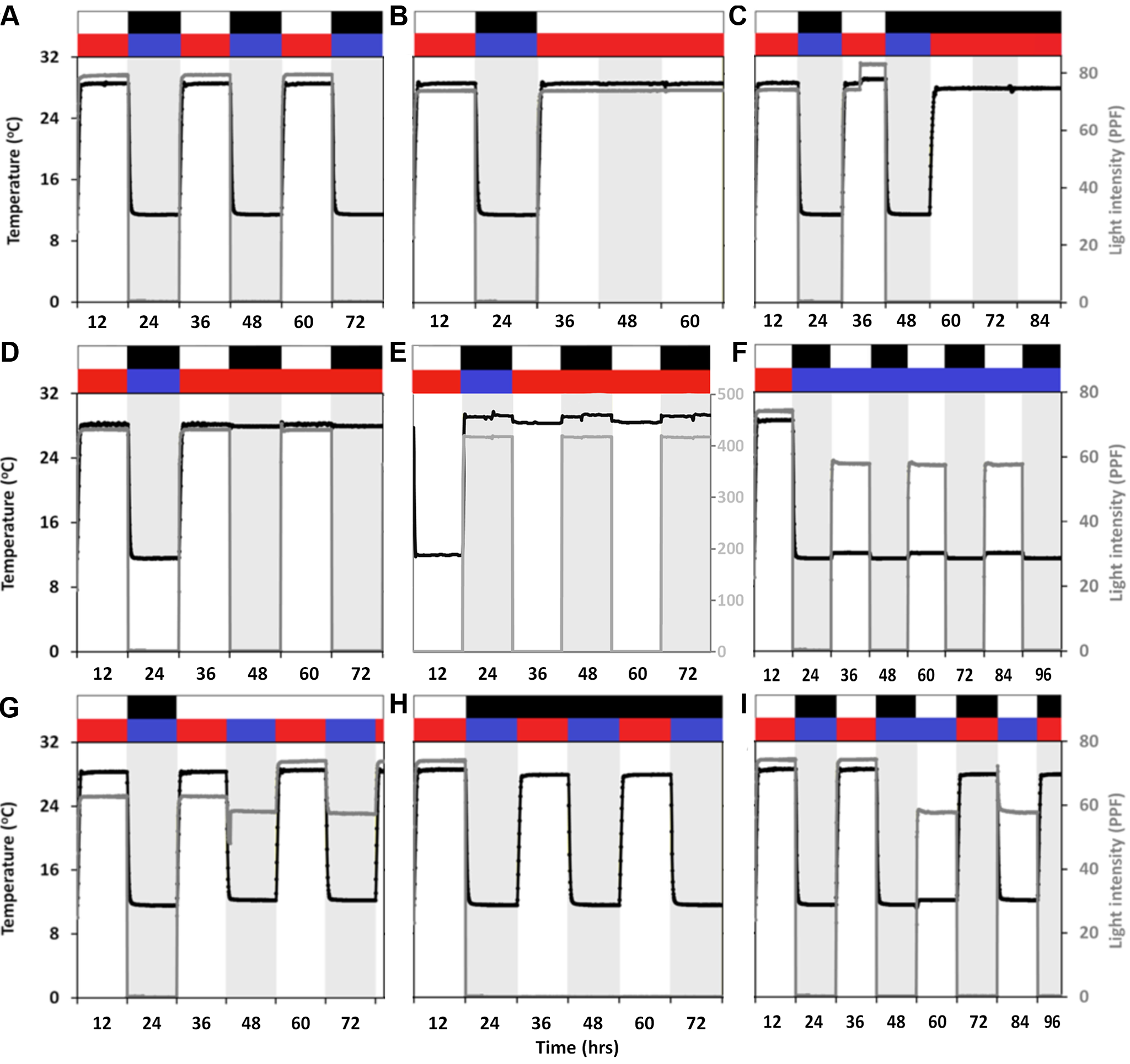

Supplement: Figure S1 — Growth chamber conditions. All plants were grown under (A) photo and thermocycles and then transferred to (B) constant 28°C light or (C) dark conditions; (D) 28°C or (F) 12°C conditions with continued photocycles; (E) 28°C conditions with continued bright light photocycles; (G) constant light or (H) dark conditions with continued thermocycles; or (I) 12°C days and 28°C nights. (TIF) [file pone.0100072.s001.tif]
